# First person – Suzie Buono and Arnaud Monseur

**DOI:** 10.1242/dmm.049681

**Published:** 2022-07-25

## Abstract

First Person is a series of interviews with the first authors of a selection of papers published in Disease Models & Mechanisms, helping early-career researchers promote themselves alongside their papers. Suzie Buono and Arnaud Monseur are co-first authors on ‘ Natural history study and statistical modeling of disease progression in a preclinical model of myotubular myopathy’, published in DMM. Suzie is a senior associate scientist in the lab of Belinda Cowling at Dynacure, Illkirch, France, investigating rare diseases, especially centronuclear myopathies. Arnaud is a senior manager of statistics and data science at Pharmalex, Mont-St-Guibert, Belgium, working on innovative clinical trials, disease modelling and Bayesian statistics.


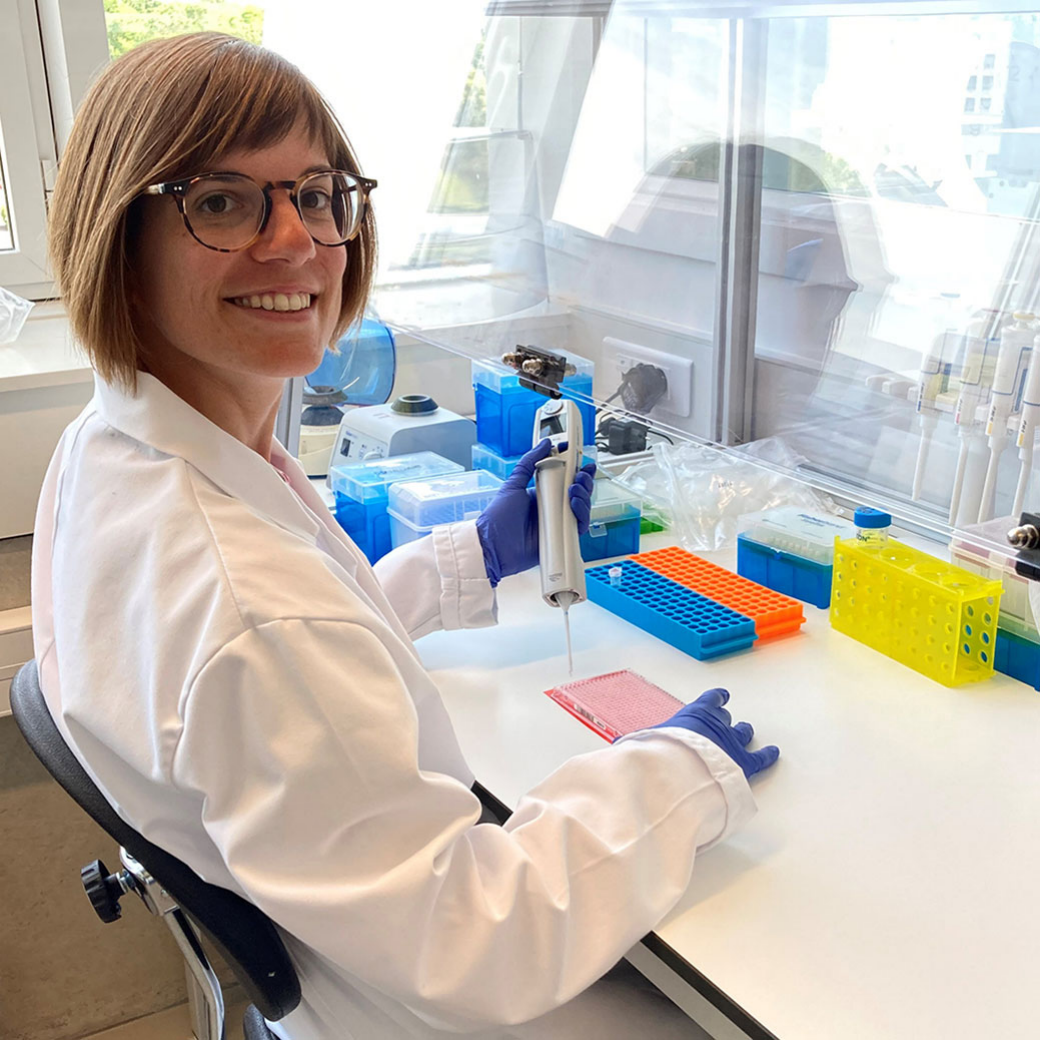


**Suzie Buono**

**How would you explain the main findings of your paper to non-scientific family and friends?**

Myotubular myopathy is a very severe neuromuscular disease affecting mainly boys (1/50,000), and no treatment is currently available. Myotubular myopathy is characterised by abnormal muscle cell organisation resulting in muscle weakness. Animal models are important to better understand human diseases, and develop therapies and biomarkers.

In this paper, we provided a better understanding of the disease progression of a mouse model, *Mtm1*^−/y^ mice, which reproduces most of the symptoms observed in patients (reduced survival, muscle weakness). We have designed a model that allows better understanding of the progression of myotubular myopathy. This model could enable researchers to detect changes from the normal progression of disease and help identify potential drugs.

**What are the potential implications of these results for your field of research?**

The study provided a description of the disease progression in myotubular myopathy mice, taking into account several parameters: survival, body weight, muscle strength and kyphosis. We also studied which parameters are the most important for determining disease progression. Finally, we tested a therapeutic approach, reducing *Dnm2*, in a dose–response study.

This study will be useful for researchers in the neuromuscular disease community using this mouse model, as we provide information on mouse behaviour across different colonies and different animal houses, and published a standard operating procedure to standardise analysis across the community.

Overall, this study could help future research in understanding the mechanism of centronuclear myopathy disease and help identify potential therapies that could reduce the symptoms as characterised by the disease progression identified herein.

**What are the main advantages and drawbacks of the model system you have used as it relates to the disease you are investigating?**

**S.B.:** The myotubular myopathy mice reproduce many symptoms seen in patients (muscle weakness, abnormal muscle cell structure) and represent an important tool to better understand the pathology, develop biomarkers to follow the progression of the disease or the treatment efficacy, and to perform proof-of-concept of therapies. However, the mice as an experimental disease model do not always translate all disease features observed in patients.

**A.M.:** This is an easy tool to understand the progression of the disease and deviations from it. The main drawback is that this is still only an animal model in mice, which needs more confirmation from further research in humans.

“The rescue of the mice by targeting *Dnm2* was impressive by observing the mice recovering and rapidly gaining force.”

**Figure:**
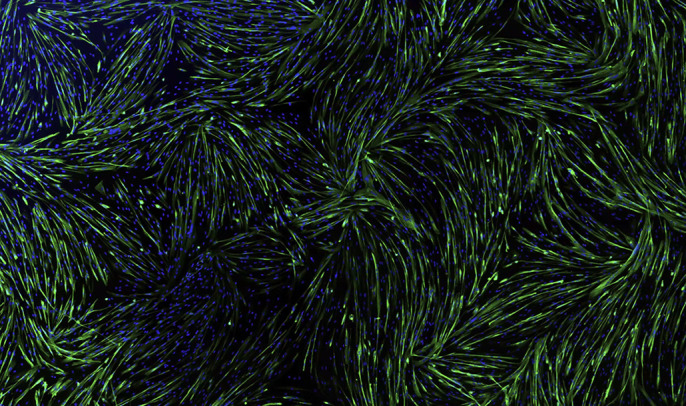
Human immortalised myoblasts were plated on coated coverslips and differentiated for 4 days in order to obtain multinucleated fibres (green, myosin heavy chain staining; blue, nuclei staining).

**What has surprised you the most while conducting your research?**

**S.B.:** What has surprised me the most while conducting my research is the reproduction and the clinical observation of the symptoms observed in myotubular myopathy in the *Mtm1*^−/y^ mice (muscle weakness, ptosis, reduced survival), as well as the quick progression of the pathology. I was also impressed by the reproducibility of the generation of this mouse model in two different animal houses, which can have an impact on the phenotyping by changing environment. This is really an advantage to help the community of researchers using this mouse model. The rescue of the mice by targeting *Dnm2* was impressive by observing the mice recovering and rapidly gaining force.

**A.M.:** I was particularly surprised that the statistical model used in the disease progression modelling, which includes survival (joint model), was able to appropriately reproduce the observed data. Furthermore, this model can easily be used for potential drug identification, which is particularly exciting.

**Describe what you think is the most significant challenge impacting your research at this time and how will this be addressed over the next 10 years?**

**S.B.:** Regarding translation to clinic, I think the most challenging is to translate a therapeutic concept from animal models to patients and the time it takes to develop a drug to test in clinic. To better understand preclinical data and the implication in human disease, promoting opportunities to encourage a close interaction between researchers and clinical experts, will be useful. Also, to continue to share standard operating procedures at the international level could help the community as it is important to standardise analysis. I think key advances/results in the research field including ‘negative’ results should be shared with the community easily to work in a collaborative manner.

**A.M.:** From a statistical point of view, I think the reproducibility issues are one of the key challenges in future research. I see the Bayesian paradigm as a solution to this as it avoids relying too heavily on *P*-values.

**What changes do you think could improve the professional lives of early-career scientists?**

To help young researchers to develop their networking with researchers could be useful to help them to decide in which field they would like to work. Organising more events between young researchers and experts in academic research, private companies, business and development could be very useful. For example, organising events focused on career development by speaking with professionals would be helpful.

**What's next for you?**

**S.B.:** The next step is to continue to focus on myopathies, to better understand the disease pathology and to develop therapies to help patients.

**A.M.:** To continue to help scientists use more advanced and innovative statistical techniques in order to better understand and tackle the challenges they face in their research.
